# Distraction plating for bilaterally severely comminuted distal radius fracture: a case report

**DOI:** 10.1080/23320885.2023.2165497

**Published:** 2023-01-11

**Authors:** Yuta Izawa, Hiroko Murakami, Tetsuya Shirakawa, Kazuo Sato, Toshiki Yoshino, Yoshihiko Tsuchida

**Affiliations:** Department of Orthopedic Trauma Center, Sapporo Higashi Tokushukai Hospital, Sapporo, Japan

**Keywords:** Distal radius fracture, open fracture, distraction plating, external fixation

## Abstract

We report a case in which distraction plating was performed for bilateral highly comminuted distal radius fractures. The upper extremities’ range of motion and function was acceptable. Thus, distraction plating can be a good option for relatively young patients with severe comminution of the radius and soft tissue damage.

## Introduction

The goal of treating distal radius fractures is to obtain a stable and movable wrist joint. Various treatment options are available, including conservative treatment, but open reduction and internal fixation are required in cases with severe instability or high disposition. The gold standard for internal fixation is volar locking plate fixation [1,2], and fragment-specific fixation is recommended when the articular surface is severely comminuted [3,4]. However, high-energy trauma may be accompanied by severe comminution and soft tissue damage, which are difficult to treat using a traditional internal fixation strategy. In such cases, external fixation is generally regarded as the next best treatment option [5,6]. External fixation spans the wrist joint continuously to maintain alignment until bone union; however, pin site infection and inconvenience owing to the fixation apparatus that the patient has to wear are common problems with this approach. Distraction plating is a method of bridging fixation from the radial shaft to the third metacarpal bone subcutaneously on the dorsal side and is used as an alternative to external fixation [7–10]. Although there is concern that the limitation of range of motion will remain due to the fixation of the wrist joint until implant removal, it has been reported that an acceptable range of motion of the wrist joint will eventually be obtained. Herein, we report a case in which distraction plating was performed for a bilateral highly comminuted distal radius fracture, with acceptable results obtained in the wrist joint’s range of motion and function.

## Case report

**Figure 1.**
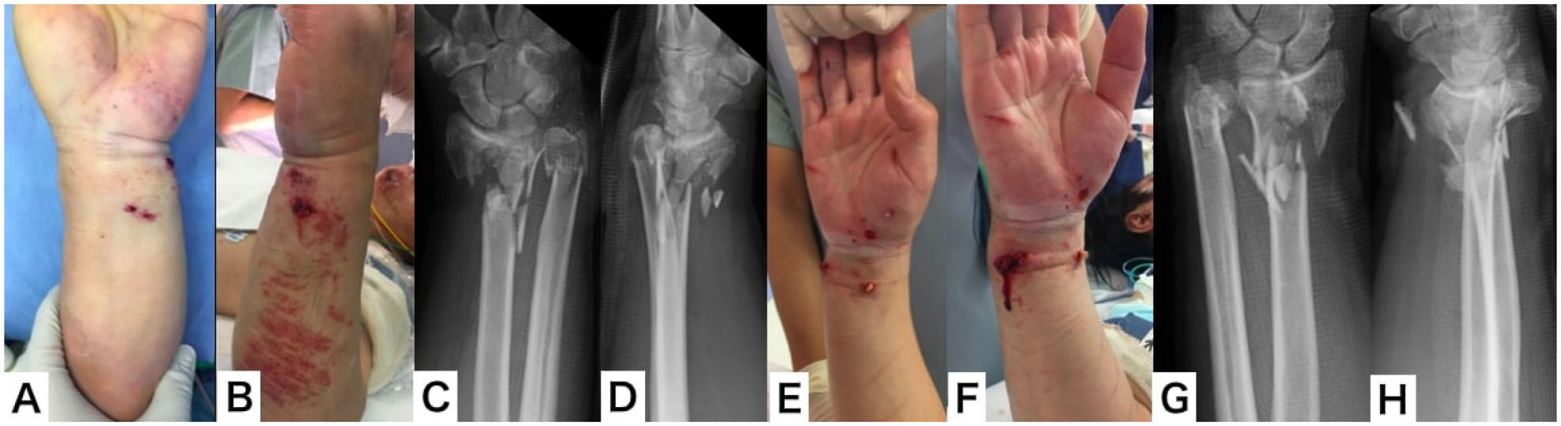
Appearance and plain x-ray on arrival at the emergency department. (A,B) Appearance of the left wrist; (C,D) plain x-ray of the left wrist; (E,F) appearance of the right wrist; (G,H) plain x-ray of the right wrist.

**Figure 2.**
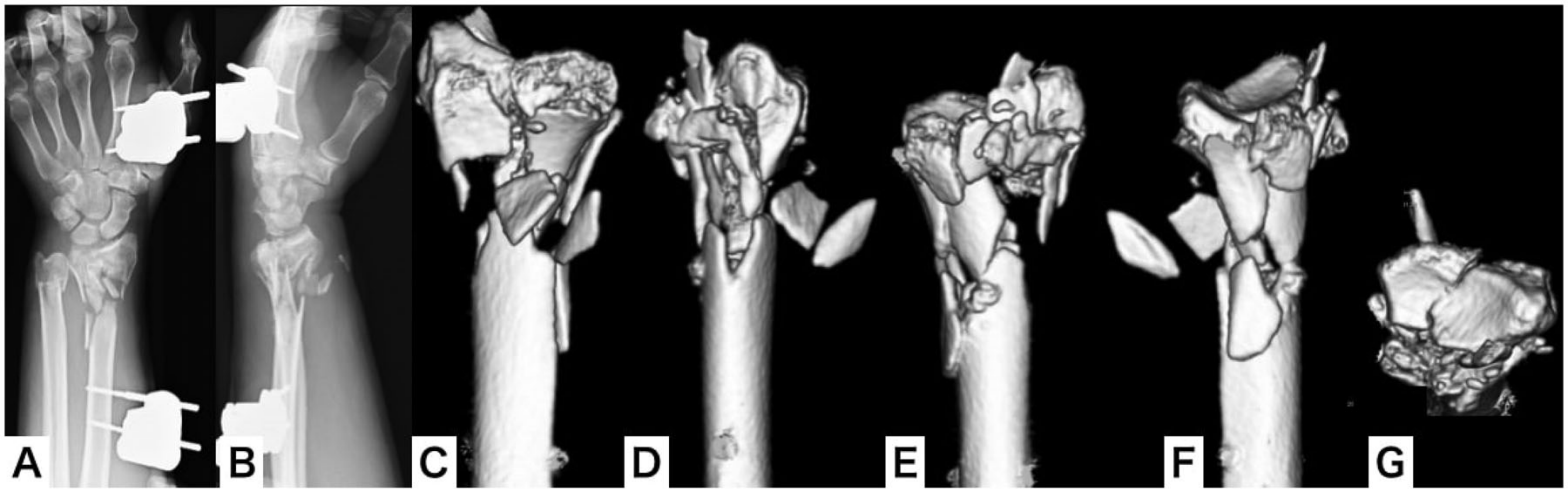
Plain x-ray and three-dimensional computed tomography (3-D CT) of the left wrist after external fixation as a first-line treatment. (A,B) Plain x-ray of the left wrist; (C–G) 3-D CT of the left wrist.

**Figure 3.**
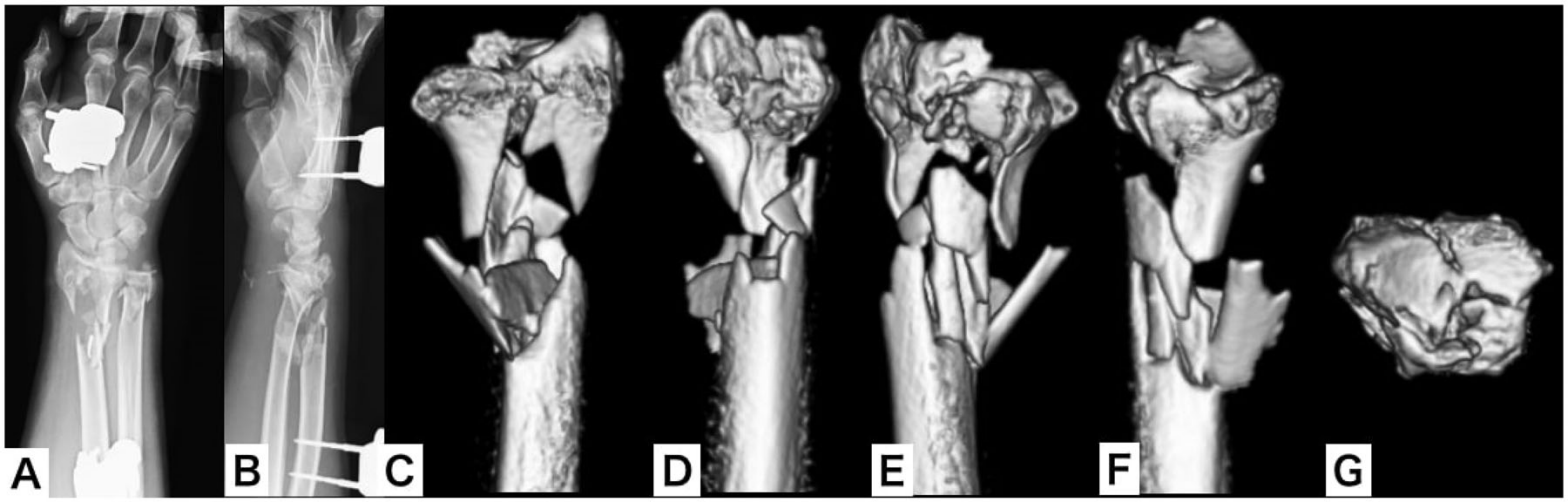
Plain x-ray and three-dimensional computed tomography of the right wrist after external fixation as a first-line treatment. (A,B) Plain x-ray of the right wrist; (C–G) 3-D CT of the right wrist.

A male patient aged 50 years jumped from the 3rd floor of his house in an attempted suicide and was injured. The patient presented at the emergency department and was diagnosed with cerebral contusions, multiple rib fractures, pulmonary contusions, and bilateral distal radial and ulnar open fractures (AO:2R3C3.3, AO:2U3A3, Gustilo-Anderson classification: type 2) (Figure 1). Irrigation, debridement, and external fixation were performed on the day of the injury. Computed tomography (CT) revealed bilateral severe comminution of the radial metaphysis (Figures 2 and 3). We evaluated that it would be difficult to perform internal fixation using a volar locking plate or fragment-specific fixation, as the soft tissue condition was also poor. Hence, we planned to fix the injuries using distraction plating.

**Figure 4.**
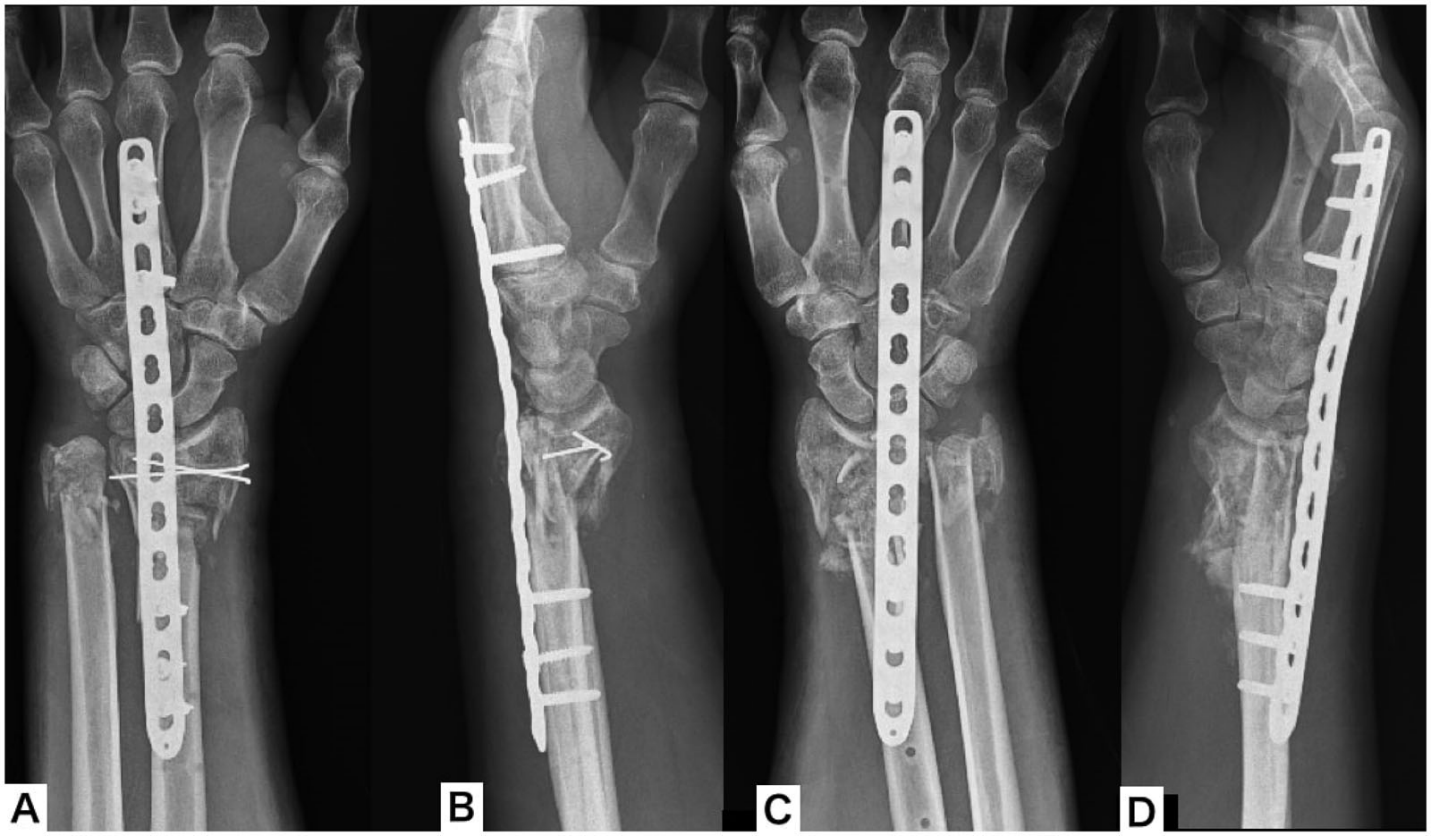
Plain x-ray of both wrists after distraction plating. (A,B) Plain x-ray of the left wrist; (C,D) plain x-ray of the right wrist.

Simultaneous bilateral surgery was performed on the 7th day after injury. First, a dorsal approach was used to open the 3rd and 4th compartments. Then, the open wound on the volar side was extended to enable manipulation of the fragments, including the articular surface. Next, a 12-hole metaphyseal plate was placed under the extensor tendons and fixed to the radial metaphysis and the third metacarpal bone with traction using an external fixator. Next, a Kirschner wire was inserted into the fragments, including the articular surface from the volar side, and reduced as a joystick. Finally, the bone defect was filled with autologous cancellous bone (Figure 4).

**Figure 5.**
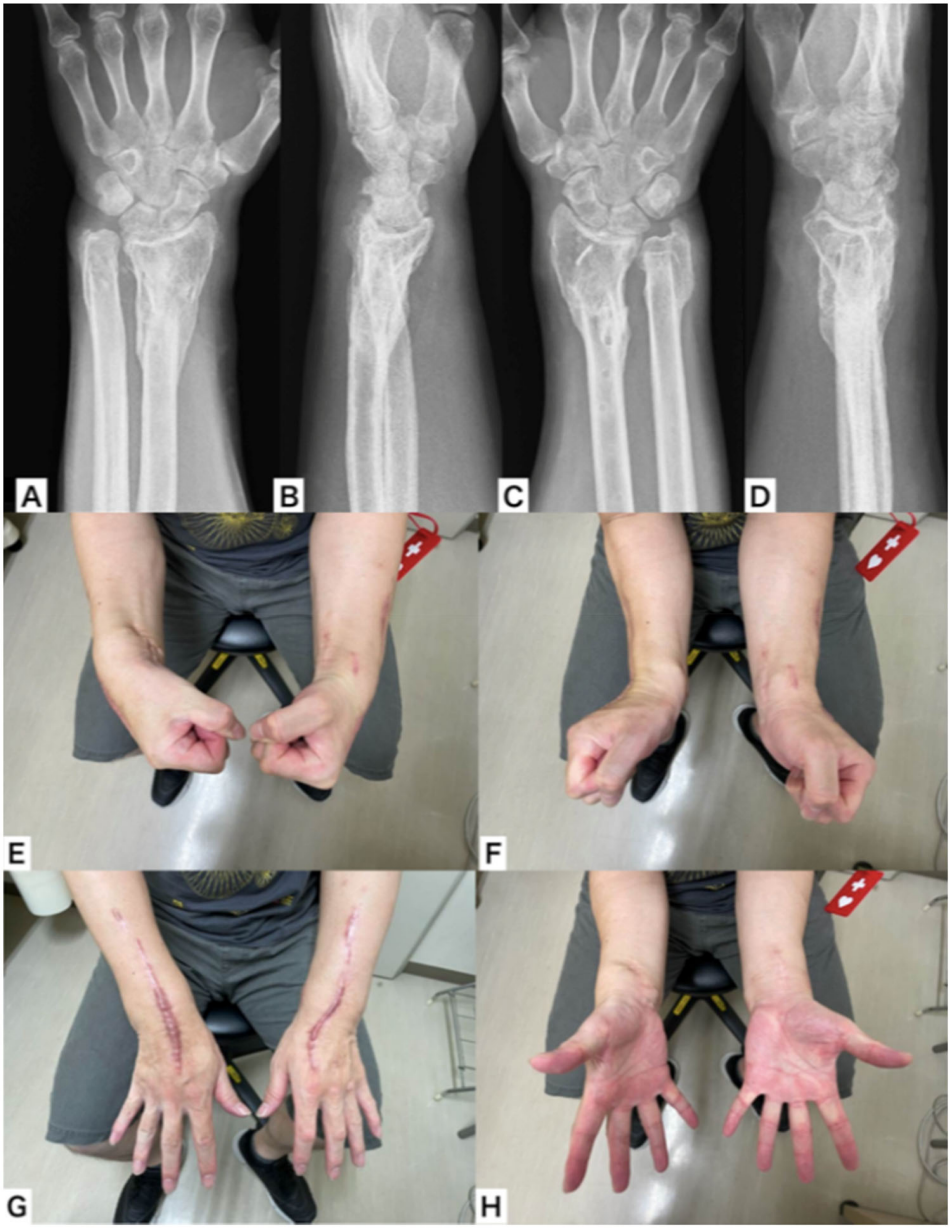
Plain x-ray, appearance, and range of motion of both wrists 1 year postoperatively. (A,B) Plain x-ray of the left wrist; (C,D) plain x-ray of the right wrist; (E) active palmar flexion; (F) active dorsiflexion; (G) forearm pronation; (H) forearm supination.

Casting was not performed after surgery; daily movement was permitted, and range of motion training of the fingers was performed immediately. Weight-bearing on both upper extremities was restricted until plate removal was performed. There were no problems with movement using the fingers during the period of wrist fixation. Three months after the operation, bone union was observed in both the radius and the ulna on plain X-ray and CT. The plate and Kirschner-wire were removed, and range of motion training of the wrist joint was started. The upper extremities’ range of motion and function 1 year following the operation were as follows. In the left upper extremity, (i) the wrist joint active palmar flexion and dorsiflexion were 35° and 60°, respectively; (ii) the grip strength was 29 kg, and pronation and supination of the forearm were 85° and 75°, respectively. In the right upper extremity, (i) the wrist joint active palmar flexion and dorsiflexion were 45° and 65°, respectively; (ii) grip strength was 24 kg, and pronation and supination of the forearm were 80° and 60°, respectively. The DASH and HAND20 scores were 5 and 4 points, respectively (Figure 5). Following recovery, the patient returned to performing manual labor without any complaints.

## Discussion

Distraction plating makes it possible to maintain the reduction position without burdening the soft tissue while waiting for bone union and can obtain radiologically and functionally acceptable results [7–10]. Moreover, distraction plating is more stable than an external fixator and has the advantage of eliminating the risk of pin-site infection [11]. However, because the wrist joint must be fixed until the plate is removed, the limitation of the wrist joint’s range of motion may remain after recovery. Therefore, it is preferentially considered an acceptable operation for fractures in older patients with severe comminution and poor soft tissue condition but is less often preferred in younger patients.

Bruke et al. who reported distraction plating for the first time applied this method to >300 cases and found it useful for severely comminuted distal radius fractures [7]. Richard et al. reported that distraction plating was performed in 33 patients with severely comminuted distal radius fractures. According to the authors, postoperative radiological parameters, range of motion, and functional score were acceptable [8]. Perlus et al. performed a literature review about distraction plating and reported that results comparable to volar locking plating were obtained for intra-articular distal radius fractures. According to their report, the average final range of motion was 47.6° for active wrist flexion, 50.5° for active wrist dorsiflexion, 74.2° for forearm pronation, and 76.0° for forearm supination, whereas grip strength was 79.2% of the healthy side [9]. Comparable range of motion and grip strength were obtained in our case. Furthermore, the fingers’ range of motion was not restricted during the treatment, regardless of whether the plate was removed. The patients achieved satisfactory function and were able to return to work.

This case involved multiple fractures due to high-energy trauma, accompanied by intracranial and trunk injuries. Therefore, it was necessary to avoid long-term surgery for both upper extremities. In addition, since the metaphysis of the radius was severely comminuted, it was difficult to fix the bone fragments sufficiently using a volar locking plate alone. Furthermore, because the soft tissue condition was poor, placing several implants by fragment-specific fixation was difficult. Therefore, distraction plating was performed on both sides simultaneously, although the patient was not older. This case is the first in the literature in which the distraction plate was fixed simultaneously on both sides. Since the fractures could be firmly fixed without causing soft tissue trouble, rehabilitation and daily activities could be started immediately after the operation. The final range of motion and functional results were acceptable, and patient satisfaction was high. These findings indicate that distraction plating may be a good option for patients who are relatively young, have severe comminution of the radius, and are concerned about the condition of soft tissues, especially for high-energy trauma injuries that require active treatment and rehabilitation for accompanying injuries.
